# Exploring the Role of Primary Care Nurses in Dietary Management for Migrants With Diabetes: A Scoping Review

**DOI:** 10.1111/jan.70208

**Published:** 2025-09-05

**Authors:** Keycee Silang, Vainess Mbuzi, Coralie Graham, Leah East

**Affiliations:** ^1^ School of Nursing and Midwifery University of Southern Queensland Toowoomba Queensland Australia; ^2^ School of Nursing and Midwifery, Centre for Health Research University of Southern Queensland Toowoomba Queensland Australia; ^3^ University of Southern Queensland. Toowoomba Queensland Australia; ^4^ School of Health University of New England Armidale New South Wales Australia

**Keywords:** cultural issues, diabetes, health promotion, migrant health, nurse roles, nurse–patient relationships, primary care

## Abstract

**Aim(s):**

To explore how primary care practitioners, including nurses, provide dietary diabetes management to migrants.

**Design:**

The scoping review followed, a refined and structured methodological framework and adhered to the Joanna Briggs Institute Scoping Review guidelines.

**Methods and Data Sources:**

Searches were conducted across CINAHL, PubMed, and Scopus databases to identify studies published between 2000 and 2024 that focus on dietary diabetes care for migrants in Primary Health Care settings. Data were synthesised using thematic analysis.

**Results:**

The search identified 377 studies, with 30 meeting the inclusion criteria. Analysis revealed four themes: (1) cultural influences on diabetes management, (2) culturally tailored dietary interventions, (3) communication challenges and (4) access and availability of diabetes care.

**Conclusion:**

Culturally competent primary care practices are crucial for effective diabetes dietary management for migrants, as they can enhance patient engagement, adherence, and overall health outcomes. Primary care nurses are uniquely positioned to address the barriers experienced by migrant populations through tailored care delivery.

**Implications for Patient Care:**

The findings provide actionable guidance for nurses to implement targeted and culturally responsive approaches in delivering dietary diabetes management, aiming to improve patient adherence and health outcomes among migrants.

**Impact:**

This review identified significant literature gaps in how primary care nurses provide culturally responsive dietary guidance for migrant patients with diabetes. The four themes identified have emphasised the need for culturally preserving care to enhance patient engagement and adherence to clinical guidance. The findings will directly impact nursing practice, education, and clinical guidelines globally, enabling nurses to deliver more effective and culturally responsive diabetes care that improves diabetes‐related disparities among migrants globally.

**Reporting Method:**

The review adhered to PRISMA‐Sc guidelines.

**Patient or Public Contribution:**

No patient or public contribution. This review received no funding from public, commercial or not‐for‐profit sectors.

**Summary**

- What is already known
  ○ Primary health care nurses are central to chronic disease management, but their role in culturally tailored dietary diabetes care for migrants remains underexplored.
  ○ Migrant patients face cultural, linguistic, and systemic barriers that affect dietary management in diabetes care.
  ○ Culturally responsive care can improve engagement and adherence among migrant patients.
- What this paper adds
  ○ Culturally tailored dietary diabetes care provided by primary health care nurses is pivotal for improving engagement, adherence, and outcomes among migrant populations.
  ○ Highlights that primary health care nurses can improve dietary adherence by adapting education to cultural food traditions, practices, and preferences.
  ○ Identifies nursing‐led strategies such as culturally adapted resources, bilingual education sessions, and community‐based partnerships to address barriers in migrant diabetes care.
- Implications for Practice/Policy
  ○ Primary health care nurses should be supported to deliver culturally preserving dietary care through responsive cultural competency training and resource provision.
  ○ Health services should embed nurse‐led, culturally responsive dietary interventions into routine primary health care diabetes management.
  ○ National and global diabetes policies should recognise and resource the pivotal role of primary health care nurses in reducing disparities for migrant populations.

## 1 Introduction

The World Health Organisation (WHO) estimates that approximately 830 million people worldwide are living with diabetes (WHO 2024). Similarly, in 2021, an estimated 1 in 20 Australians (just over 1.3 million) were living with Type 1 and Type 2 diagnosed diabetes (Australian Institute of Health and Welfare 2024). However, diabetes prevalence is not uniform across population groups, with migrant populations showing a notably higher burden of disease. Research indicates that migrants exhibit significantly higher diabetes prevalence, with rates in some groups more than double those of Australian‐born individuals (Abouzeid et al. 2013). Globally, the burden of diabetes is also consistently higher among migrants than native‐born populations, as observed in regions such as Europe, Oceania, and America (Montesi et al. 2016; Rawal et al. 2021). The onset of diabetes, particularly Type 2 diabetes mellitus (T2DM), often occurs 5–10 years earlier in migrant populations, with increased incidence of chronic complications (Montesi et al. 2016).

The disproportionate burden of diabetes (Type 1 and 2) among migrants highlights the need for tailored dietary management that is culturally relevant and adaptable to diverse settings. Primary Health Care (PHC) practitioners, particularly nurses, are well‐positioned worldwide to deliver culturally responsive diabetes care due to their continuous patient relationships, community‐based roles, and accessibility. However, the role of PHC nurses in delivering this care remains underexplored globally, despite shared challenges across health systems. This scoping review aims to synthesise literature on PHC practices in dietary diabetes management for migrants. The review seeks to identify key facilitators and barriers in PHC practice to optimise PHC dietary management among migrant populations. The findings have potential applicability across global contexts with diverse populations, offering transferable strategies for strengthening culturally responsive PHC nursing practice.

## 2 Background

Effective dietary management is a cornerstone of diabetes care, as it can enhance glycaemic control, reduce reliance on medications, and lower the risk of diabetes‐related complications (Evert et al. 2019). Literature underscores key dietary management that promotes the intake of vegetables, fruits, whole grains, lean protein, and legumes (Forouhi et al. 2018). In contrast, the intake of processed foods, particularly refined grains and red meat, has been found to elevate insulin and glucose responses, increasing the risk of pancreatic stress and Type 2 diabetes (Kim et al. 2016).

Research on a low‐carbohydrate diet for diabetes management is plentiful, with consensus showing significant improvement in diabetes‐related biometrics such as HbA1c, fasting glucose, and triglyceride levels (Salvia and Quatromoni 2023). The importance of macronutrient regulation in diabetes management is well recognised, with literature suggesting that optimising the intake of other macronutrients (e.g., protein, fat, fibre) to meet a patient's individual energy needs can positively influence glucose homeostasis, lipid profile, and body composition (Gupta et al. 2018).

Although the role of dietary management in diabetes care is widely acknowledged, migrant populations face unique challenges in adhering to dietary recommendations due to the complex interplay of social, cultural, and linguistic factors that influence their health habits. Migrants often struggle with cultural food preservation while adjusting to new dietary norms in their host country, as traditional diets often consist of high carbohydrate and sugar content (Li‐Geng et al. 2020). This traditional diet is often in conflict with Western diabetes dietary guidelines, posing a significant obstacle to adherence within migrant populations (Kim 2023).

A key socio‐cultural factor is the role of food in social comfort and enjoyment, with communal eating and family gatherings as cultural norms (Li‐Geng et al. 2020). The dietary modifications required for those with diabetes can complicate social interactions and family dynamics (Kim 2023). Dietary modifications can create a sense of isolation during shared or communal meals, as patients with diabetes may feel excluded or worry about disrupting traditional food practices. Adhering to dietary modifications can also impose emotional stress, with patients expressing their concerns with being “a burden” to their families (Li‐Geng et al. 2020). The disconnection from cultural traditions can result in loss of cultural identity, which impacts emotional and social well‐being. In addition to cultural challenges, migrants also encounter barriers related to low English proficiency and health literacy, and limited access to services and healthier alternatives (Li‐Geng et al. 2020). This further complicates navigating healthcare systems, leading to suboptimal health seeking behaviours and increased risk of diabetes complications (Rizqillah and Ma'rifah 2020).

As migration continues to rise globally, the need for culturally preserving healthcare becomes increasingly pivotal. Pettersson et al. (2023) found that the nutrition guidance provided to migrants often lacks cultural sensitivity, resulting in poor adherence and ineffective self‐care practices. Research indicates that migrants are more likely to adhere to dietary recommendations that promote cultural identity preservation (Kohinor et al. 2011). Given the critical role of culturally competent diabetes care, PHC nurses are uniquely positioned to deliver dietary guidance that facilitates cultural preservation.

While the involvement of general practitioners and dietitians in diabetes care is well established, the role of PHC nurses remains underexplored (Farzaei et al. 2023). PHC nurses are pivotal in chronic disease management due to their regular interactions with primary care patients. However, little is known regarding the strategies they implement to tailor dietary counselling to the varying cultural needs of migrants. The role of PHC nurses extends beyond clinical duties, also encompassing patient education, health promotion, and assisting patients with navigating self‐management practices (Alshammari et al. 2021). Given the limited understanding of the role of PHC nurses in delivering dietary diabetes care to migrant populations, a scoping review was undertaken to explore the existing literature.

## 3 The Review

### 3.1 Aim

The aim of this study was to examine the existing literature on how PHC practitioners, particularly nurses, deliver dietary diabetes management for migrant populations. The review seeks to identify current management, facilitators, and barriers to practice, and other socio‐cultural factors that impact dietary care for migrants with diabetes.

### 3.2 Design

The scoping review was conducted following the framework established by Arksey and O'Malley (2005) and further refined by Levac et al. (2010). Additionally, the Joanna Briggs Institute (JBI) Scoping Review guidelines (Peters et al. 2020) were adhered to throughout the review process. The research question guiding this review was “How do Primary Health Care (PHC) practitioners, particularly nurses, deliver dietary management to migrant populations with diabetes?”. No protocol was registered.

### 3.3 Search Methods

Following the JBI Evidence Synthesis framework (Peters et al. 2020), a comprehensive literature search was conducted in June 2024 using three databases: CINAHL, PubMed, and Scopus. The initial search specifically focused on identifying literature that examined the role of primary care nurses in delivering dietary diabetes management to migrant populations. However, due to the limited literature yielded from the initial search focused on primary care nurses, the inclusion criteria were broadened to include studies involving other PHC practitioners to capture a more comprehensive understanding of current PHC diabetes practice.

The search strategy was informed by the Population, Concept, and Context (PCC) framework outlined by the JBI (Peters et al. 2020). From the initial search of titles and abstracts, a list of key terms and Medical Subject Headings (MeSH) was compiled. Terms related to migrant populations included “migrant*”, “immigrant*”, “foreign*”, “transients and migrants” [MeSH], “emigrants and immigrants” [MeSH], “ESL” and “English as a second language”. The concept terms focused on diabetes and dietary care, such as “diabetes”, “diabetes mellitus” [MeSH], “diabetic*”, “diabetes management” and “management of diabetes”. Context terms included “primary care”, “primary health care” [MeSH], “practice nurse” and “community care”. Terms related to the provider role focused on nursing, including “nurse”, “nurses”, “nursing” and “nurses” [MeSH].

Boolean operators (AND, OR) and truncation were used to combine terms across concepts. For example, a representative search string in PubMed included: (“Nurses”[Mesh] OR nurse* OR nursing) AND (“Diabetes Mellitus”[Mesh] OR diabetes OR diabetic*) AND (“Transients and Migrants”[Mesh] OR migrant* OR immigrant* OR foreign* OR ESL OR “English as a second language”). In CINAHL, both subject headings and keyword terms were used similarly. In Scopus, the search was conducted using both indexed subject terms and by scanning the title, abstract, and keywords to ensure comprehensive coverage of relevant studies. The search was limited to studies published in English between 2000 and 2024. Only peer‐reviewed journal articles, including qualitative, quantitative, and mixed‐methods studies, as well as systematic and scoping reviews, were included. The detailed search terms and strategy are outlined in Appendix A.

### 3.4 Inclusion and Exclusion Criteria

The inclusion criteria for this review were: (1) migrant participants over 18 years old diagnosed with DM, as diabetes care for paediatric patients differs significantly from adult care due to developmental, physiological and psychosocial factors unique to children and adolescents (American Diabetes Association 2021) (2) studies focusing on dietary management or strategies aimed at improving diabetes management through dietary management, (3) studies conducted in PHC or community care settings where PHC practitioners provide care, (4) peer‐reviewed qualitative and quantitative studies, original research, systematic reviews, scoping reviews, (5) studies published in English, and (6) studies published between 2000 and 2024. This timeframe was chosen to capture research conducted within modern primary health care models, which developed after the transition from comprehensive to selective PHC approaches described by Cueto (2004). Limiting the review to studies published from 2000 onwards ensures relevance to current clinical and policy contexts.

The exclusion criteria included (1) studies focusing on patients diagnosed with conditions other than DM Type 1 or Type 2, as gestational and other forms of diabetes involve distinct clinical pathways and dietary management approaches (Rasmussen et al. 2020), (2) studies that did not mention dietary management for diabetes management, (3) dietary management delivered outside of primary care settings, and (4) studies that did not include migrant populations as part of the sample.

### 3.5 Search Outcome

**FIGURE 1:**
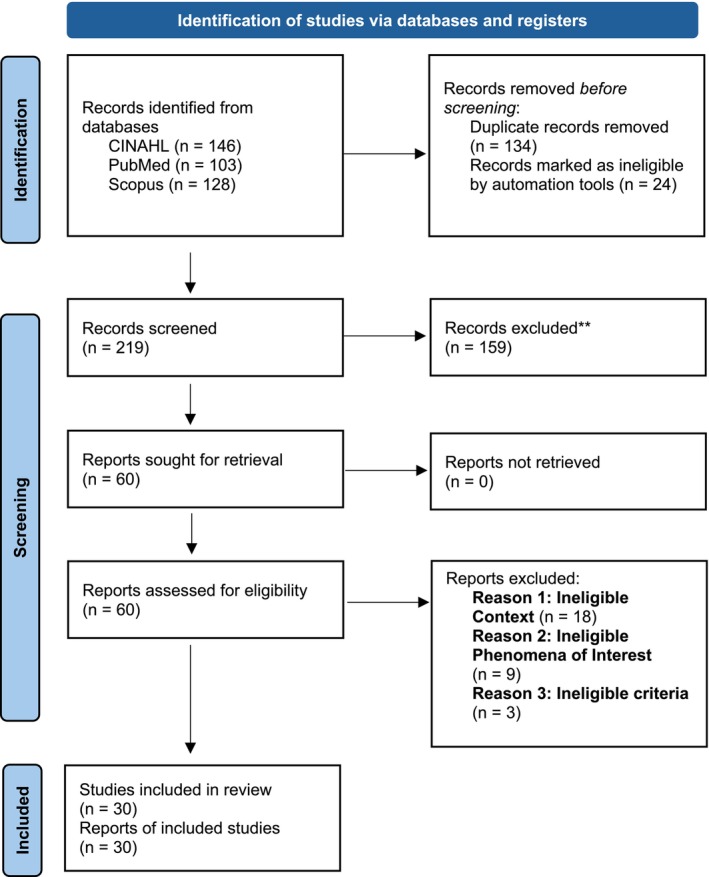
PRISMA diagram.

The selection process, illustrated in Figure 1, involved screening of 377 potential studies from across CINAHL (*n* = 146), PubMed (*n* = 103), and Scopus (*n* = 128). After removing 134 duplicate records and 24 records marked as ineligible by another deduplication automation tool, 219 studies were screened. Titles and abstracts were independently reviewed by three members of the research team, resulting in the exclusion of 159 studies based on irrelevance to the research question. The remaining 60 studies were sought for full‐text retrieval, and no studies were left unretrieved. Following the review of these 60 studies, 29 studies were excluded for the following reasons: 18 studies were excluded due to an ineligible context, 9 due to an ineligible phenomenon of interest, and 3 for failing to meet the eligibility criteria outlined in the PCC framework. As detailed in Figure 1, the selection process resulted in 30 studies meeting the inclusion criteria and included in the final review.

### 3.6 Quality Appraisal

Although scoping reviews do not traditionally focus on appraising the quality of studies (Arksey and O'Malley 2005), literature has emphasised the importance of integrating a critical appraisal process to enhance the transparency and trustworthiness of a review (Levac et al. 2010). For this review, the JBI Critical Appraisal Tools were employed to assess the methodological quality of the qualitative, quantitative, and systematic review studies, while mixed‐methods studies were critically appraised using the Mixed Methods Appraisal Tool (MMAT). All studies scored 70% or higher on the quality indicators and were included in the review (See Tables B1, B2, B3, B4: Appendix B).

### 3.7 Data Abstraction

One researcher extracted and summarised data following the data extraction guide outlined with the JBI methodology guidelines (Pollock et al. 2023). The characteristics of the included studies, presented in Table B5, capture critical details from each study, including the author, year of publication, aim and study methods, participants, location and setting, results and key findings from the study. Two separate reviewers independently validated the extracted data to ensure the accuracy of the findings.

### 3.8 Synthesis

Thematic analysis was adopted to identify and summarise key findings from the studies (Naeem et al. 2023). Thematic analysis provides a flexible yet structured framework to synthesise and extract data that can be modified to suit different study designs and data types (Nowell et al. 2017). The studies in the review were systematically analysed by two researchers to minimise bias and ensure robustness of the analysis. Initially, the included studies were reviewed to extract similarities and patterns used to code into preliminary themes. Then, the researchers compared and discussed their codes until a consensus was reached on the themes. Ultimately, the thematic analysis of the studies revealed four key themes, including the impact of culture on diabetes management, culturally tailored management for diabetes management, communication in diabetes care, and access and availability of diabetes management among migrants.

## 4 Results

The general characteristics of the included studies are outlined in Table B5. Studies were conducted across various geographical regions, with the highest number originating from the United States (*n* = 9), followed by the United Kingdom (*n* = 4), Sweden (*n* = 4), Australia (*n* = 3), and Canada (*n* = 3). Other countries represented include Israel (*n* = 1), Ireland (*n* = 1), Italy (*n* = 1), and Finland (*n* = 1). The study designs encompassed qualitative (*n* = 18), quantitative (*n* = 5), mixed methods (*n* = 3), and systematic or scoping reviews (*n* = 5), which reflect a diversity of approaches to understanding dietary diabetes management among migrant populations. Most studies focused on culturally tailored management and communication strategies in diabetes care, while several others examined the influence of culture on diabetes self‐management practices and healthcare access within the context of dietary diabetes care. Methodological quality appraisal using the relevant JBI or MMAT tools (See Tables B1, B2, B3, B4: Appendix B) indicated that all studies were of moderate to high methodological rigour, with clear objectives, sound study designs, and appropriate recruitment and data collection processes. Common weaknesses varied by study designs, including limitations in cultural congruity and participant voice representation in some qualitative studies, gaps in baseline comparability and participant allocation clarity in some quantitative studies, and absence of risk‐of‐bias assessment in review studies. These quality considerations are referenced within each theme and considered in the interpretation of findings.

An analysis of the included studies identified four key themes: the impact of culture on diabetes management practices, culturally tailored diabetes management, communication in diabetes care, and access to care for migrant populations. These themes reflect the key focus areas for improving dietary diabetes care delivery for migrant groups.

### 4.1 Impact of Culture on Diabetes Management Practices

The theme of cultural impact on diabetes management emerged as the overarching factor that influences dietary practices among migrant populations. This theme reflects the findings from 10 included studies (see Table B5) and highlights how cultural practices, values, and beliefs impact migrants' ability to adhere to clinical dietary recommendations and diabetes self‐management practices. Most of the studies contributing to this theme were qualitative (See Table B1: Appendix B) and demonstrated strong methodological rigour, with clear aims and robust designs and data collection. Some had gaps in cultural congruity (Q6) and representation of participants' voices (Q7), indicating that while cultural issues were explored, they were not always fully embedded in the research design. The mixed‐methods studies contributing to this theme (See Table B4: Appendix B) have met all critical appraisal criteria, strengthening the reliability of these findings.

Mu'Min Chowdhury et al. (2000) examined how religious dietary laws and traditional food classification among British Bangladeshis influenced their food preferences, with a focus on halal foods and categorising foods by “strength” and “digestibility” rather than nutritional value. These culturally grounded preferences often led to food practices that are misaligned with clinical dietary guidelines for diabetes management (Mu'Min Chowdhury et al. 2000). For example, with this cultural food classification, plants growing from the ground are perceived as weak due to incomplete extraction of the earth's nourishing power, while red meat is regarded as stronger than other sources like poultry and fish. This classification is associated with low vegetable consumption and high red meat intake, which do not align with clinical dietary recommendations that prioritise vegetables, whole grains, and lean meats for managing diabetes (Kim et al. 2016).

Similarly, Wan et al. (2023) highlighted that high‐carbohydrate dietary traditions and social stigma posed significant barriers to diabetes dietary adherence, while noting that participants valued culturally aligned support from health professionals of similar ethnic backgrounds. The findings by Dor‐On et al. (2022) also highlighted the cultural conflict faced by migrants in balancing traditional values with adherence to dietary guidelines for diabetes. The participants' collectivist values often led them to prioritise social obligations, such as accepting food offered by hosts, even when it conflicted with dietary management advice (Dor‐On et al. 2022).

As identified by Roth et al. (2021), the impact of culture is evident in the experiences of Sudanese migrants in Australia, who struggled to adapt to dietary advice due to the significance of traditional carbohydrate‐rich foods that are central to their social gatherings. The collectivist nature of the participants, characterised by prioritising social obligations and communal values over individual needs, also led them to prioritise social expectations over dietary restrictions (Roth et al. 2021). Many participants relied on traditional remedies such as cinnamon tea, fenugreek seeds, mahogany bark, and Arabic gum to lower blood sugar levels, which can conflict with clinical recommendations (Roth et al. 2021).

The impact of collectivism on diabetes management is reinforced by Leung et al. (2014), who found that participants are reluctant to disrupt cultural norms, such as sharing traditional high‐carbohydrate meals with family. The study also emphasised that participants showed a preference for group‐based diabetes education and valued shared experiences and peer interactions. A barrier identified by Leung et al. (2014) was the lack of culturally relevant diabetes information accessible to participants. The lack of culturally relevant health information, particularly dietary recommendations, negatively influenced participants' uptake of health advice (Leung et al. 2014).

On the other hand, Ethiopian migrants, for example, viewed diabetes as “punishment” rather than a result of lifestyle choices and genetics, which complicates self‐management practices (Dor‐On et al. 2022). This study also highlighted that PHC nurses play a key role in addressing cultural and language gaps by facilitating peer models in group sessions and using professional liaisons fluent in the participants' native language to support the sessions. During group sessions, peer models who shared their successful diabetes management journeys acted as motivational figures for other participants, which reduced scepticism about clinical dietary recommendations (e.g., reduced sugar and fat intake) and feelings of social and cultural isolation (Dor‐On et al. 2022). Unlike other PHC professionals like GPs, who may only provide brief consultations, or dietitians who focus on specific dietary plans, PHC nurses are uniquely positioned to facilitate peer models through group education and utilise professional language liaisons to support the sessions. The participants reported that the professional liaisons provided clearer explanations than their family members (Dor‐On et al. 2022).

The desire to avoid being burdensome was also evident in migrant cultural values, which influenced individuals' self‐management of diabetic practices (Leung et al. 2014). For example, Leung et al. (2014) found that Chinese immigrants displayed high regard for authority and upheld a passive patient role, which created barriers in communication between their health provider and limited their engagement in active self‐management. The participants perceived the role of doctors and nurses as responsible for treatment rather than patient education, which negatively impacted their capacity to ask their health providers for diabetes education and relied on friends and family for sources of diabetes management advice (Leung et al. 2014). The tendency to seek diabetes information through personal networks was also identified in the study conducted by Hyman et al. (2014). The study identified that migrants are more likely to engage with PHC nurses and community‐based services to obtain diabetes management guidance compared to hospital‐based care (Hyman et al. 2014). PHC nurses, especially those working in the community, were perceived as the main source of education and regular health monitoring among the participants (Hyman et al. 2014).

Despite the challenges of implementing dietary modifications, Roth et al. (2021) found that primary care providers, including diabetes educators, were viewed as trusted sources for diabetes education, especially in the early phase of diagnosis. Likewise, the study conducted by Staff et al. (2017) highlighted the preference among migrants to receive culturally adapted care, with PHC nurses providing support and tailored education to address individual needs. However, the study found that migrants are less likely to participate in standard diabetes programmes than native‐born populations, yet culturally tailored interventions and management were explicitly requested by migrant patients (Staff et al. 2017).

### 4.2 Culturally Tailored Management for Diabetes Management Among Migrants

The theme of culturally tailored management emerged from studies highlighting the improved health outcomes associated with culturally adapted interventions. Of the 30 studies reviewed, 10 specifically examined culturally tailored management, emphasising its role in enhancing engagement, adherence, and health outcomes among migrant populations. The quantitative studies contributing to this theme (See Table B2: Appendix B) generally demonstrated high‐quality intervention design and clear outcome measurements. Some had weaknesses in participant allocation (Q2) and baseline comparability (Q4), while the reviews (See Table B3: Appendix B) provided a comprehensive synthesis of findings but lacked an explicit risk‐of‐bias assessment (Q9). The qualitative studies were predominantly high quality, with minimal gaps in cultural congruity.

One example, Kim et al. (2009) found that a culturally tailored self‐help intervention for T2DM management, including a 6‐week psycho‐behavioural education programme by bilingual nurses and a nutritionist, home glucose monitoring with tele‐transmission, and 24 weeks of monthly bilingual nurse counselling, helped achieve HbA1c target levels and improved diabetes management skills. At 18 weeks, 10% of participants achieved their target HbA1c levels of less than 7%, which increased to 15.5% by 30 weeks after the intervention commenced (Kim et al. 2009). The participants also reported high satisfaction with the intervention, with a 95.2% retention rate, improved quality of life and reduced depressive symptoms (Kim et al. 2009). This is consistent with the findings in Choi and Rush (2012), which revealed that diabetes sessions delivered by a bilingual nurse and subsequent follow‐up resulted in significant improvements in HbA1c, HDL levels and other related biometrics (e.g., waist circumference) among migrant participants. Delivering sessions in the participants' native language and discussing dietary diabetes management using traditional Korean foods improved engagement and helped participants understand the application of dietary recommendations to their own cultural practices (Choi and Rush 2012). This reinforces that while dietitians may provide specific dietary plans, it is often nurses who ensure the translation of dietary advice into daily practice through continuous patient contact and integration of cultural identity into ongoing management. In a systematic review, Joo (2014) found similar improvements across Asian immigrant populations, linking culturally tailored diabetes programmes such as bilingual services and resources, community‐based delivery, family involvement, to improved clinical outcomes and patient satisfaction.

Similarly, addressing the emotional and spiritual needs of Haitian migrants has been found to be vital to improving self‐management diabetic practices among this population (Magny‐Normilus et al. 2019). The participants expressed the need for healthcare providers, who understand their customs and food preferences, as a lack of cultural understanding affects their adherence to self‐management practices (Magny‐Normilus et al. 2019). A review conducted by Osokpo et al. (2021) identified that culturally tailored diabetes management, which is aligned with migrants' cultural practices, improves adherence to self‐care practices. Osokpo et al. (2021) detailed that migrants are more likely to engage with diabetes services that incorporate traditional remedies and uphold their cultural identity and beliefs. Gulyani et al. (2024) also identified that culturally appropriate diabetes management strategies and patient‐provider relationships serve as facilitators to optimal diabetes management. Migration‐related factors, such as language barriers and financial constraints, along with tradition‐based practices like communal eating, are significant barriers for South Asian migrants (Gulyani et al. 2024). These findings are consistent with the broader themes of integrating cultural considerations into dietary diabetes management for migrant populations.

The study by Pettersson et al. (2023) affirmed that a culturally appropriate digital tool can promote diabetes self‐management in migrants. The tool is a digital platform designed to support self‐care in migrant patients with T2DM by providing accessible, multilingual educational resources and practical guidance on diabetes management (Pettersson et al. 2023). Pettersson et al. (2023) emphasised that integrating suitable language and considering cultural context when developing a digital tool is vital to improving self‐care adherence and diabetes control in the target population. This is congruent with the findings of Caselgrandi et al. (2016), who demonstrated that the delivery of diabetes care to Ghanian migrants, facilitated by a culturally competent Clinical Research Nurse (CRN), led to increased engagement with healthcare services among the Ghanian community in Italy. The CRN played both a supportive and advocacy role by acting as a liaison between participants and the healthcare team, while organising follow‐ups with participants to assess their perceptions of care and health outcomes (Caselgrandi et al. 2016). This advocacy role reflects a broader nursing function that is distinct from other PHC providers, where the nurse serves as a consistent patient advocate to achieve cultural preservation. The study by McEwen et al. (2010) examined the role of a bilingual and bicultural Certified Diabetes Educator (CDE) and a nurse researcher in developing a culturally tailored intervention for Mexican Americans with Type 2 diabetes living in the US‐Mexico border region. The CDE and nurse researcher collaboratively designed the six‐monthly group sessions and home visits, which were delivered by the CDE and trained promotoras (community health workers). Findings from the study indicated an increase in self‐management efficacy and a decrease in diabetes‐related distress and sedentary behaviours as a result of the culturally tailored interventions. Fleming and Gillibrand (2008) also emphasised the importance of personalising diabetes care and integrating complementary and biomedical management, urging nurses to consider individual interpretations of culture rather than broad stereotypes to address socio‐economic and contextual barriers to effective healthcare delivery.

### 4.3 Communication in Diabetes Care for Migrant Populations

The theme of communication barriers emerged from studies highlighting the critical role of language and culturally tailored communication in diabetes management. Of the thirty studies included, six focused specifically on communication barriers, examining the impact on patient‐provider relationships, self‐management, and access to effective diabetes care. These were primarily informed by qualitative studies (See Table B1: Appendix B) and a quantitative study, several of which met all critical appraisal criteria and demonstrated strong methodological rigour.

Martin (2014) found that migrant patients often struggled to articulate their health concerns in their second language, leaving them feeling overwhelmed and misunderstood. In the study, providers were found to prioritise biomedical metrics (e.g., hbA1c, BSL) and disregarded the “lifeworld” concerns from patients (Martin 2014). For example, a patient emphasised the significance of honey in their diet but was dismissed by providers without meaningful engagement. Patterns of perspectives misalignment persisted over a five‐month period across consultations with different providers (Martin 2014). Similarly, Brämberg et al. (2012) found that the Diabetes Nurse Specialist (DNS) assumed a dominant role in patient interactions, characterised by one‐way communication and a reliance on medical care checklists. It was also observed that the DNS failed to respond to the patient's social context and concerns and merely provided care that was task‐focused (Brämberg et al. 2012). These findings highlight the role variation even within PHC nursing, underscoring the need for nurses to move beyond task‐based care and embrace their unique relational capacity, which differs from other PHC professionals.

Another study by Rhodes and Nocon (2003) explored the use of informal interpreters like family members within a Bangladeshi community in the UK. While this practice facilitates basic communication, it often leads to miscommunication and misinterpretation of critical health information (Rhodes and Nocon 2003). Findings also indicated that the reliance on family members for communication fostered dependency and limited patients' ability to actively partake in the care planning and discussion (Rhodes and Nocon 2003).

The impact of effective and culturally tailored communication is further evident in Rechenberg et al.'s (2021) study, which focused on implementing a language‐concordant health coaching for Latinx migrants who had diabetes. The program involved Spanish‐speaking nurse‐led bi‐weekly sessions to help participants set personal health goals, identify barriers, and develop confidence to achieve their goals. Participants received up to six bi‐weekly calls to discuss experiences and challenges related to self‐management, including dietary modifications, medication adherence, and exercise (Rechenberg et al. 2021). The results showed improvement in glycaemic control and emotional well‐being in the intervention group compared to the control group. This nurse‐led intervention illustrates how the nursing role differs from GPs in diabetes management in PHC, highlighting that nurses are essential for ongoing patient‐provider communication, motivational coaching, and culturally preserving monitoring.

Alzubaidi et al. (2016) highlighted a similar need for culturally appropriate communication and ongoing support for Arabic‐speaking migrants. Alzubaidi et al. (2016) emphasised the value of a storytelling method, which involves discussing experiences and learning from shared narratives to help reinforce self‐management behaviours within a culturally familiar context. The participants reported that storytelling sessions felt more relevant to their cultural context and helped them address diabetes management barriers, particularly around dietary and lifestyle modifications. Alzubaidi et al. (2016) noted the lack of continuous support and communication from primary care providers beyond the initial diagnosis, leading to gaps in knowledge and impaired self‐management skills. Without regular clinical guidance, participants reported feelings of isolation, confusion, and overwhelm, which contributed to poor treatment adherence (Alzubaidi et al. 2016). Similarly, Choi et al. (2018) underscore the importance of culturally tailored communication in diabetes education for Chinese patients, where directive and prescriptive communication styles were preferred over participatory approaches. While Alzubaidi et al. (2016) demonstrated the effectiveness of storytelling to contextualise self‐management practices within cultural narratives, Choi et al. (2018) revealed that Chinese patients' cultural values of hierarchy and compliance often resulted in passive engagement during consultations. Both studies highlight that misaligned communication approaches can lead to confusion and disengagement among participants.

### 4.4 Access and Availability of Diabetes Care for Migrants

The theme of access and availability emerged from studies that examined structural, cultural, and personal barriers faced by migrants when engaging with diabetes services. Among the thirty studies, five specifically focused on how the structural, cultural, and personal barriers shape access and utilisation of diabetes care. This theme was informed by qualitative, review, and mixed‐methods studies. The qualitative studies (See Table B1: Appendix B) were generally robust, with some studies showing gaps in cultural congruity and participant voice representation, potentially limiting cultural contextualisation. The contributing mixed‐method studies met all criteria, offering strong integration of qualitative and quantitative perspectives.

An example by Joo and Lee (2016) identified that cost, language barriers, and limited access to culturally appropriate diabetes services hinder diabetes management among Korean‐American older immigrants. These perceived barriers have been found to reduce their engagement and uptake of services, resulting in ineffective diabetes management and health outcomes (Joo and Lee 2016). The findings from Joo and Lee (2016) highlight that family and peer support significantly improve access and availability of diabetes care for migrant populations. Participants emphasised that family members assist with key diabetes‐related tasks, such as organising medical appointments and managing medications. For example, a female participant noted that her daughter's daily support was crucial in managing her diabetes, illustrating the importance of culturally familiar support networks in enhancing care access.

The recurring themes of access and availability of diabetes care were also present in Berterö and Hjelm (2010), which highlighted systemic barriers to diabetes care such as delays in receiving care, communication issues, lack of support from healthcare providers, and economic challenges. The study also found that navigating an unfamiliar health system and differences in provider expectations create challenges for migrants, directly impacting their access to diabetes care. Similarly, Hadziabdic et al. (2020) noted that migrants with diabetes often faced challenges with accessing linguistically and culturally appropriate diabetes education. It was demonstrated that migrants have lower knowledge about their condition compared to native populations (Hadziabdic et al. 2020). Whilst the role of the multi‐disciplinary diabetes care team was emphasised in the study, findings indicated that many primary care clinics lack culturally appropriate resources and availability of interpreters for migrants (Hadziabdic et al. 2020). In such contexts, nurses become the point of continuity, supporting patients to navigate and access interpreters or community resources. This advocacy and navigation support role distinguishes nursing from other PHC practitioners.

## 5 Discussion

This scoping review examined how PHC professionals, particularly nurses, deliver dietary management for diabetes among migrant populations. The review provided a nuanced understanding of the facilitators and barriers that impact the effectiveness of dietary diabetes care among migrants, highlighting opportunities to address improved health outcomes. The analysis identified four key themes: cultural influences on diabetes management, culturally tailored interventions, communication barriers, and access to care. The themes underscore the complex interplay of cultural, social, and structural factors shaping diabetes outcomes in migrant communities and highlight the central role of culturally responsive nursing practice.

Language barriers, limited health literacy, and unfamiliarity with healthcare systems significantly hinder migrants' engagement with diabetes services (Rasi 2020). PHC nurses are well positioned to bridge these gaps through bilingual communication, interpreter services, and culturally resonant strategies such as storytelling and group education (Leung et al. 2014; Hyman et al. 2014). These approaches not only enhance understanding but also foster trust and inclusivity, enabling patients to engage more actively in their care (Alzubaidi et al. 2016; Magny‐Normilus et al. 2019). Culturally relevant communication and education through group sessions and peer support programmes, led by PHC nurses, can strengthen social networks and provide emotional and behavioural support in culturally meaningful ways (Fisher et al. 2012; Kolomvotsou and Riza 2021; Tang et al. 2015). Storytelling and language‐concordant coaching further contextualise clinical advice, making it more relatable and actionable. These strategies empower patients and promote sustained behavioural change (Alzubaidi et al. 2016; Rechenberg et al. 2021).

Integrating cultural food preferences into dietary guidance is another critical strategy. Dietitians often provide the technical framework, but PHC nurses have the opportunity to adapt these frameworks into practical and culturally responsive management during continuous patient interactions. Many migrants retain traditional diets high in carbohydrates or starches, which often conflict with Western dietary recommendations (Kindarara et al. 2017). When clinical advice disregards cultural practices, it can lead to dissonance, mistrust, and poor adherence (Cha et al. 2012; Chowdhury et al. 2000; Dor‐On et al. 2022; Wan et al. 2023). PHC nurses can address this by adapting dietary education to include culturally appropriate substitutions and modifications, preserving cultural identity while supporting health goals (Gulyani et al. 2024; Osokpo et al. 2021).

Cultural beliefs also shape perceptions of illness and influence self‐management. For example, viewing diabetes as a form of punishment, as seen among Ethiopian migrants, may lead to reliance on traditional remedies over clinical advice (Dor‐On et al. 2022). These beliefs can hinder adherence to clinical dietary advice, as they drive reliance on traditional remedies that align more closely with cultural values but conflict with evidence‐based recommendations (Roth et al., 2021; Leung et al. 2014; Hjelm and Bard 2013). While traditional remedies serve as a means of preserving cultural identity, their misalignment with clinical guidelines can compromise glycaemic control and worsen health outcomes (Shewamene et al. 2021). PHC nurses can navigate these beliefs through respectful, patient‐centred dialogue, offering culturally acceptable alternatives and fostering therapeutic relationships that support self‐management (Saenz et al. 2024).

As trusted providers, PHC nurses are uniquely positioned to deliver personalised, culturally responsive care, combining relational continuity with patients, accessibility and health education. Their regular patient interactions enable them to address cultural barriers, advocate for patient needs, and integrate traditional practices with evidence‐based care (Lukewich et al. 2022). High patient satisfaction with nurse‐led chronic disease management further supports this approach (Halcomb et al. 2015).

To meet the diverse needs of migrant populations, PHC nurses must be equipped with cultural competency training. This includes understanding traditional health beliefs, communication styles, and dietary customs. Such training enhances care quality, builds trust, and improves patient engagement (Gulyani et al. 2024; Kaihlanen et al. 2020). Embedding cultural competency training into nursing education and professional development is essential for delivering inclusive, effective dietary interventions.

Despite the insights gained, gaps remain in understanding how PHC nurses integrate cultural preservation with evidence‐based dietary management. Further research is needed to inform clinical practice and develop frameworks that support culturally tailored care in diverse migrant populations. While this review provides valuable insights into dietary management for migrants in PHC settings, several limitations must be acknowledged. First, the included studies often lacked reporting on participant demographics, particularly regarding cultural, socio‐economic, and educational diversity within migrant sub‐groups. This limits the ability to contextualise findings across different populations and highlights the need for more targeted research (Malmusi et al. 2010). Second, inconsistencies in how PHC roles were defined across studies reduce the generalisability of the findings. Finally, the exclusion of non‐English publications may have restricted the inclusion of perspectives from broader and more diverse populations, potentially introducing language bias.

## 6 Conclusion

This scoping review synthesised the current literature on dietary practices employed by PHC professionals, particularly nurses, in managing diabetes among migrant populations. Findings reveal key opportunities to strengthen PHC nursing practice by embedding cultural preservation into dietary care models, improving access to linguistically and culturally appropriate resources, and addressing systemic barriers that hinder culturally responsive care delivery. Key themes underscore the importance of cultural preservation and facilitating management that addresses the complexities of migrants' lived experiences, including linguistic, social, and systemic challenges. The findings highlight that PHC nurses should be supported through targeted cultural competency training, resource allocation, and policy collaboration to and deliver culturally preserving dietary care. National and global strategies must recognise the critical role of PHC nurses in diabetes management for migrants and provide adequate systemic support and resources to enable nurses to deliver inclusive care. This review calls for further research exploring intervention‐based studies that test culturally preserving dietary models in PHC settings, evaluating their impact on health outcomes and assessing applicability across diverse migrant populations and health systems.

## Conflicts of Interest

The authors declare no conflicts of interest.

## Data Availability

The authors have nothing to report.

## Appendix A Search Strategy Table

**Table:**

Key concept #1	Key concept #2	Key concept #3	Key concept #4	Date range	Limiters?
**#9** **Nurses** Nurse OR Nurses OR Nursing **Not Used due to low results:** “health profession*” OR “community health”	**#10** **“Primary Health Care”[Mesh]** “primary healthcare” OR “primary health care” OR “practice nurs*” OR “primary care”	**#11** **“Diabetes Mellitus”[Mesh]** Diabetes OR Diabetic* “diabetes management” OR “management of diabetes” **Not Used due to low results:** Management OR Diet* OR intervention	**#12** **“Transients and Migrants”[Mesh] OR “Emigrants and Immigrants”[Mesh]** Migrant* OR Immigrant* OR ESL OR “english as second language”	2000—present	**Language:** English **Choose which types of resource will be included**: Journal articles (peer‐reviewed) Books/chapters Theses Conference proceedings Government reports Organisational reports Business reports Newspaper articles

Abbreviations: PCC, population, concept, context; PICO, population, intervention/interest, context, outcome.

### CINAHL Search

((MH Nurses+) OR (Nurse OR Nurses OR nursing OR “health profession*”)) AND ((MH “Diabetes Mellitus+”) OR (Diabetes OR Diabetic*)) AND ((MH “Migrants” OR MH “Immigrants+” OR MH “Emigration and Immigration+”) OR (Migrant* OR Immigrant* OR foreign* OR ESL OR “english as second language”)).

### PubMed Search

(((“Nurses”[Mesh]) OR (Nurse[Text Word] OR Nurses[Text Word] OR nursing[Text Word] OR “health profession*”[Text Word])) AND ((“Diabetes Mellitus”[Mesh]) OR (Diabetes[Text Word] OR Diabetic*[Text Word]))) AND ((“Transients and Migrants”[Mesh] OR “Emigrants and Immigrants”[Mesh]) OR (Migrant*[Text Word] OR Immigrant*[Text Word] OR foreign*[Text Word] OR ESL[Text Word] OR “english as second language”[Text Word])).

### Scopus Search

(((INDEXTERMS (nurses)) OR (TITLE‐ABS‐KEY (nurse) OR TITLE‐ABS‐KEY (nurses) OR TITLE‐ABS‐KEY (nursing) OR TITLE‐ABS‐KEY (“health profession*”))) AND ((INDEXTERMS (“Diabetes Mellitus”)) OR (TITLE‐ABS‐KEY (diabetes) OR TITLE‐ABS‐KEY (diabetic*)))) AND ((INDEXTERMS (“Transients and Migrants”) OR INDEXTERMS (“Emigrants and Immigrants”)) OR (TITLE‐ABS‐KEY (migrant*) OR TITLE‐ABS‐KEY (immigrant*) OR TITLE‐ABS‐KEY (foreign*) OR TITLE‐ABS‐KEY (esl) OR TITLE‐ABS‐KEY (“english as second language”))).

## Appendix B

**TABLE B1:** Critical appraisal results for included studies using the JBI‐Qualitative Critical Appraisal Checklist.

Study	Q1	Q2	Q3	Q4	Q5	Q6	Q7	Q8	Q9	Q10
Martin (2014)	Y	Y	Y	Y	Y	Y	Y	Y	Y	Y
Rhodes and Nocon (2003)	Y	Y	Y	Y	Y	U	N	Y	U	Y
Amirehsani (2010)	Y	Y	Y	Y	Y	U	N	N	NA	Y
Caselgrandi et al. (2016)	Y	Y	Y	Y	Y	U	Y	Y	Y	Y
Pettersson et al. (2023)	Y	Y	Y	Y	Y	U	Y	Y	Y	Y
Alzubaidi et al. (2016)	Y	Y	Y	Y	Y	Y	Y	Y	Y	Y
Mu'Min Chowdhury et al. (2000)	Y	Y	Y	Y	Y	Y	Y	Y	U	Y
Choi et al. (2018)	Y	Y	Y	Y	Y	Y	Y	Y	Y	Y
Dor‐On et al. (2022)	Y	Y	Y	Y	Y	N	U	Y	Y	Y
Nekouei Marvi Langari et al. (2024)	Y	Y	Y	Y	Y	N	Y	Y	Y	Y
Roth et al. (2021)	Y	Y	Y	Y	Y	N	N	Y	Y	Y
Leung et al. (2014)	Y	Y	Y	Y	Y	N	N	Y	Y	Y
Hjelm and Bard (2013)	Y	Y	Y	Y	Y	N	N	Y	Y	Y
Joo and Lee (2016)	Y	Y	Y	Y	Y	N	N	Y	Y	Y
Brämberg et al. (2012)	Y	Y	Y	Y	Y	N	N	Y	Y	Y
Choi et al. (2017)	Y	Y	Y	Y	Y	Y	Y	Y	Y	Y
Magny‐Normilus et al. (2019)	Y	Y	Y	Y	Y	Y	Y	Y	Y	Y

Abbreviations: N, no; NA, not applicable; U, unclear; Y, yes.

**TABLE B2:** Critical appraisal results for included studies using the JBI Quasi‐experimental studies.

Study	Q1	Q2	Q3	Q4	Q5	Q6	Q7	Q8	Q9
Hyman et al. (2014)	Y	Y	Y	Y	N	NA	Y	Y	Y
Choi and Rush (2012)	Y	N	Y	N	Y	Y	Y	Y	Y
Rechenberg et al. (2021)	Y	Y	Y	Y	Y	Y	Y	Y	Y
Kim et al. (2009)	Y	Y	Y	Y	Y	Y	Y	Y	Y
McEwen et al. (2010)	Y	Y	Y	N	Y	Y	Y	Y	Y

Abbreviations: N, no; NA, not applicable; U, unclear; Y, yes.

**TABLE B3:** Critical appraisal results for included studies using the JBI‐systematic reviews and research syntheses.

Study	Q1	Q2	Q3	Q4	Q5	Q6	Q7	Q8	Q9	Q10	Q11
Gulyani et al. (2024)	Y	Y	Y	Y	Y	Y	Y	Y	N	Y	Y
Fleming and Gillibrand (2008)	Y	Y	Y	Y	Y	Y	Y	Y	N	Y	Y
Joo (2014)	Y	Y	Y	Y	Y	N	N	Y	N	Y	Y
Osokpo et al. (2021)	Y	Y	Y	Y	Y	Y	Y	Y	N	Y	Y
Hadziabdic et al. (2020)	Y	Y	Y	Y	Y	N	N	Y	N	Y	Y

Abbreviations: N, no; NA, not applicable; U, unclear; Y, yes.

**TABLE B4:** Critical appraisal results for included studies using the Mixed Methods Appraisal Tool Version 2018.

Study	Q1	Q2	Q3	Q4	Q5
Berterö and Hjelm (2010)	Y	Y	Y	Y	Y
Wan et al. (2023)	Y	Y	Y	Y	Y
Staff et al. (2017)	Y	Y	Y	Y	Y

Abbreviations: C, can't tell; N, no; Y, yes.

**TABLE B5:** Data extraction table for Scoping Review.

Author and title	Aim	Method	Participants	Setting	Results	Conclusion
Martin (2014) “Well I don't know what to say to that”: Exploring tensions between the voices of medicine and the lifeworld in the management of diabetes	Explore tensions between medical professionals and immigrant patient Focus on diabetes management and communication challenges	Qualitative Microanalysis of patient‐provider interactions Data collection over five months through several consultations with different providers	Single Case study on 42‐year‐old Egyptian female patient with poorly controlled Type 1 diabetes who interacts with nine healthcare providers	Ireland Diabetes clinic in an Irish hospital	Misalignment between patient's lifeworld and medical advice Communication barriers due to cultural and language differences Patient frustration due to lack of empathy and understanding	Cultural and linguistic differences hinder effective diabetes management Providers' limited engagement with patient's emotional and social context Need for improved cultural competence in healthcare
Hyman et al. (2014) Self‐management, health service use and information seeking for diabetes care among recent immigrants in Toronto	Explore self‐management practices, health service use, and information‐seeking behaviours among Black Caribbean immigrants with type 2 diabetes in Toronto	Quantitative Cross‐sectional study with structured questionnaires Participants: 48 Black Caribbean immigrants and 54 Canadian‐born individuals, aged 35–64 Recruited from community health centres, diabetes education centres, hospitals, and immigrant‐serving organisations Focus on comparing diabetes self‐management and health service use between groups	48 Black Caribbean immigrants and 54 Canadian‐born participants Age range: 35–64 years	Canada Greater Toronto Area	Black Caribbean immigrants more likely to engage in recommended diabetes self‐management practices Higher use of community health centres and allied health professionals by Black Caribbean immigrants No significant differences in regular foot exams between groups	Community health centres play a crucial role in the favourable diabetes self‐management profile of Black Caribbean immigrants The need for further research to confirm these findings and explore the role of community health centres in other immigrant communities
Gulyani et al. (2024) Barriers and facilitators of lifestyle management among adult South Asian migrants living with chronic diseases: A mixed‐methods systematic review	Synthesise studies on barriers and facilitators of lifestyle management among South Asian migrants with chronic diseases	Mixed‐methods systematic review Data were thematically analysed using the Theoretical Domains Framework (TDF)	37 studies included, mostly from the UK, Canada, USA, and Norway Total of 1559 participants from South Asian countries (India, Pakistan, Bangladesh, etc.)	Studies conducted in primary care, community settings, and hospitals Studies done in numerous countries	Identified four overarching themes: 1. Patient‐provider interaction and relationship 2. Impact of migration 3. Heritage‐based practices 4. Chronic disease management strategies	Migration and cultural practices significantly influence chronic disease management Culturally tailored interventions are needed
Rhodes and Nocon (2003) A problem of communication? Diabetes care among Bangladeshi people in Bradford‌	Explore communication issues in diabetes care among Bangladeshi people in Bradford	Qualitative Study conducted in‐depth interviews with 12 Bangladeshi people Investigated the use of family members as informal interpreters during medical consultations Interviews were mostly conducted in Sylheti and analysed using a framework approach with the assistance of software	12 Bangladeshi participants aged between 43 and 75 years, including both men and women, who had been diagnosed with diabetes for varying lengths of time	United Kingdom Study took place in Bradford, UK, focusing on the Bangladeshi community within the city	Family members often helped with interpreting, but this sometimes led to poor care due to miscommunication and dependency	study suggests the need for better communication strategies and professional interpreting services to improve diabetes care
Amirehsani (2010) Mexican Americans With Type 2 Diabetes in an Emerging Latino Community: Evaluation of Health Disparity Factors and Interventions‌	Evaluate health disparity factors and interventions for Mexican Americans with type 2 diabetes in North Carolina	Qualitative Reviewed existing literature and interventions, focusing on culturally competent diabetes care	Mexican Americans with type 2 diabetes in North Carolina	USA Emerging Latino community in North Carolina	Culturally tailored interventions improve diabetes knowledge and control but need more research in emerging communities	Culturally competent care is essential for better diabetes outcomes in Mexican American communities
Fleming and Gillibrand (2008) An Exploration of Culture, Diabetes, and Nursing in the South Asian Community	Explore how culture influences diabetes self‐management in the South Asian community	Systematic review of qualitative studies Analysed 11 qualitative studies on South Asian culture and diabetes	South Asian individuals from India, Pakistan, Bangladesh, and Nepal Mixed religious backgrounds: Muslim, Hindu, Christian, Sikh	Studies mainly conducted in the United Kingdom, with one in India	Culture is dynamic and individualised, not a rigid framework South Asians negotiate cultural and contextual factors in diabetes self‐management	Culturally appropriate nursing care should be individualised
Caselgrandi et al. (2016) Clinical Research Nurse involvement to foster a community‐based transcultural research in RODAM European study	Explore and describe the involvement of a Clinical Research Nurse (CRN) in the Italian site of the RODAM study, focusing on transcultural nursing in the Ghanaian community	Qualitative CRN managed the project with a transcultural approach, collaborating with mediators, organising activities, and promoting healthcare among the Ghanaian community	Ghanaian migrants residing in Italy, involved in the RODAM study on obesity and type 2 diabetes	Italy Conducted within the Ghanaian community in Italy, utilising Ghanaian churches as key locations	CRN's involvement led to the empowerment of the Ghanaian community, improved healthcare awareness, and participation in the study	CRN's strategies, including a transcultural approach and community engagement, were crucial in achieving study success and fostering community empowerment
Choi and Rush (2012) Effect of a Short‐Duration, Culturally Tailored, Community‐Based Diabetes Self‐management Intervention for Korean Immigrants‌	Assess the effectiveness, feasibility, and acceptability of a short‐duration, culturally tailored diabetes self‐management program for Korean immigrants	Quantitative 2‐session diabetes education program delivered by a bilingual nurse practitioner at a community centre, with follow‐up at 3 months	41 Korean immigrants with type 2 diabetes, aged 30–87	USA Korean community centre in a West Coast city	Significant improvements in A1C, HDL levels, waist circumference, and diabetes self‐care behaviours (e.g., feet checks)	culturally tailored approach in community settings can improve diabetes outcomes among Korean immigrants
Pettersson et al. (2023) Developing a Culturally Appropriate Tool to Support Self‐Care in Migrants with Type 2 Diabetes – A Co‐Design Study‌	Develop a culturally appropriate tool to support self‐care in migrants with type 2 diabetes	Qualitative Co‐design process with stakeholders, including patients, healthcare providers, and researchers, involving brainstorming and prototyping	14 migrant patients, 10 healthcare providers, 4 researchers	Sweden Primary healthcare centre in southeast, Sweden.	Developed a digital platform in Arabic and Swedish for diabetes self‐care, including text, videos, and links to resources	The co‐designed platform supports diabetes self‐care in Middle Eastern migrants, offering a culturally tailored approach
Alzubaidi et al. (2016) Time to question diabetes self‐management support for Arabic‐speaking migrants: exploring a new model of care	Explore a new model of diabetes self‐management support for Arabic‐speaking migrants	Qualitative Semi‐structured interviews and focus groups with Arabic‐speaking migrants; thematic analysis	60 Arabic‐speaking migrants with type 2 diabetes in Melbourne, Australia.	Australia Primary, secondary, and tertiary healthcare settings in Melbourne	No ongoing support beyond initial diagnosis; strong preference for face‐to‐face storytelling in gender‐specific groups	Urgent need for culturally tailored, story‐based self‐management support for Arabic‐speaking migrants
Rechenberg et al. (2021) Feasibility and Acceptability of a Language‐Concordant Health Coaching Intervention Delivered by Nurses for Latinx with Type 2 Diabetes	Test the feasibility and acceptability of language‐concordant health coaching for Latinx immigrants with type 2 diabetes	Quantitative Pilot study; random assignment to control or intervention group. Intervention group received six bi‐weekly coaching sessions in Spanish	17 Latinx immigrants with type 2 diabetes and limited English proficiency	USA Federally qualified health centres	Significant reduction in A1c, depression, and anxiety symptoms in the intervention group	Language‐concordant health coaching is feasible, acceptable, and may improve key diabetes outcomes in Latinx populations
Mu'Min Chowdhury et al. (2000) Food beliefs and practices among British Bangladeshis with diabetes: Implications for health education	Understand food beliefs and practices among British Bangladeshis with diabetes	Qualitative Unstructured narrative interviews, pile sorting of food items, and participant observation	40 first‐generation adult immigrants from Sylhet, Bangladesh, with diabetes Equal gender mix, aged 24–78	United Kingdom Inner‐city areas of Tower Hamlets, Newham, and Islington in London, UK	Strong religious and cultural food practices, resistance to dietary changes Food classified as “strong/weak” and “digestible/indigestible,” not by Western nutritional standards	Health education should respect religious and cultural food practices Tailored advice considering cultural meanings can improve dietary management
Choi et al. (2018) Diabetes management in a foreign land: A case study on Chinese Australians	Understand the experience of Chinese migrants living with type 2 diabetes in Australia Explore their culturally specific diabetes management needs and expectations	Qualitative Case study approach using participant‐observations and qualitative interviews across Melbourne and Sydney Thematic analysis with pattern matching was used for data analysis	18 participant‐observations and 12 interviews Participants included Chinese migrants with diabetes and health professionals	Diabetes education sessions and clinical sites in Melbourne and Sydney, Australia	Barriers to accessing Australian diabetes care due to language and cultural differences Chinese patients relied heavily on peer networks rather than health professionals for diabetes management Diabetes services often mismatched Chinese cultural expectations	Redesigning diabetes care services to align with collectivist principles may better support Chinese migrants in managing diabetes effectively
Dor‐On et al. (2022) Ethiopian immigrants with diabetes in Israel: Oscillating between two narratives	Examine the role of cultural differences in managing diabetes among Ethiopian immigrants living in Israel	Qualitative Semi‐structured interviews Thematic analysis focusing on participants' cultural and personal narratives regarding diabetes	16 Ethiopian immigrants with type 1 or 2 diabetes, living in Israel Mean age was 52.4 years, with a nearly equal gender split.	Israel Conducted at three Clalit Health Services general medicine clinics	Participants oscillated between familiar (Ethiopian) and foreign (Israeli) narratives in understanding and managing diabetes Challenges included conflicting views on the causes of diabetes, food practices, and trust in medical treatment versus traditional beliefs	Ethiopian immigrants in Israel face internal conflicts between traditional and modern perspectives on diabetes management Culturally competent care and involving community liaisons can improve diabetes management in this population
Joo (2014) Effectiveness of Culturally Tailored Diabetes Interventions for Asian Immigrants to the United States	Evaluate the effectiveness of culturally tailored diabetes interventions for Asian immigrants in the U.S.	Systematic review of 9 studies on culturally tailored diabetes programs for Asian immigrants	Asian immigrants with type 2 diabetes in the U.S.	USA Community‐based settings in the U.S., including California, Hawaii, New York, and Maryland	Culturally tailored programs improved clinical outcomes (e.g., A1C levels) and psycho‐behavioural outcomes, with high patient satisfaction	Culturally tailored diabetes interventions are effective for Asian immigrants, but more rigorous studies are needed to confirm these findings
Osokpo et al. (2021) Maintaining cultural identity: A systematic mixed studies review of cultural influences on the self‐care of African immigrants living with non‐communicable disease‌	Identify cultural factors influencing self‐care among African immigrants with chronic illnesses	Systematic mixed studies review of nine studies on African immigrants' self‐care practices	African immigrants with non‐communicable diseases living outside Africa	Studies conducted in the U.S., Australia, Sweden, the Netherlands, and the UK	Cultural norms, non‐Western illness interpretations, and structural challenges influence self‐care. Family support aids self‐care but cultural identity can both help and hinder it	Cultural and structural factors shape self‐care behaviours in African immigrants. Integrating cultural considerations into healthcare is essential for improving self‐care
Nekouei MarvLangari et al. (2024) Registered nurses' perceptions of healthy lifestyle counselling for immigrants in primary healthcare: A focus group study‌	Explore registered nurses' perceptions of healthy lifestyle counselling for immigrants to prevent chronic diseases	Qualitative Focus group interviews with 23 registered nurses	23 registered nurses working in primary healthcare across four Finnish municipalities	Finland Primary healthcare settings in Finland (Espoo, Vantaa, Lappeenranta, Joensuu)	Participant perceptions were related to uniform counselling practices, challenges in counselling immigrants, the need for culturally tailored services and utilising insights from practical experience to improve the counselling service	Culturally sensitive health promotion services are essential to improve immigrant health outcomes
Kim et al. (2009) A community‐based, culturally tailored behavioural intervention for Korean‐Americans with Type 2 diabetes	Test the efficacy of a culturally tailored diabetes management intervention for Korean‐American immigrants (KAIs)	Quantitative Randomised controlled trial with 79 KAIs, involving a 6‐week education program, home glucose monitoring, and bilingual nurse counselling	79 Korean‐American immigrants with uncontrolled type 2 diabetes in the Baltimore‐Washington area	USA Community‐based settings in the Baltimore‐Washington area	Significant reduction in hbA1C levels and improvements in psychosocial outcomes for the intervention group	The culturally tailored intervention effectively improved glucose control and diabetes management skills among KAIs
Roth et al. (2021) Knowledge and perceptions around self‐management of type 2 diabetes among a Sudanese community in Australia: a qualitative study	Explore the perceptions and experiences of diabetes self‐management among Sudanese migrants in Australia	Qualitative Qualitative study with semi‐structured in‐depth interviews of 12 Sudanese participants in Melbourne, Australia	12 Sudanese immigrants living with type 2 diabetes, who were recruited at GP clinics, community centres	Australia Interviews conducted in mutually agreed public location in Melbourne, Australia	Barriers: Language differences, burden of self‐management, traditional dietary practices, and social obligations Enablers: Positive relationships with health professionals, support networks, religion, and traditional remedies	Cultural factors, including traditional practices and familial obligations, significantly influence diabetes management in the Sudanese community. Culturally appropriate health services are needed
Berterö and Hjelm (2010) Social support as described by foreign‐born persons diagnosed with type 2 diabetes mellitus and living in Sweden‌	Explore and describe the meaning of social support and its impact on the life situation of foreign‐born persons with type 2 diabetes in Sweden	Mixed‐methods study using semi‐structured interviews and the Norbeck Social Support Questionnaire (NSSQ)	34 foreign‐born adults with type 2 diabetes, aged 36–73 years Predominantly from the Middle East and former Yugoslavia	Sweden Conducted at a hospital‐based diabetes clinic and primary healthcare centres	Social support was perceived as limited, particularly around the time of diagnosis Support needs varied by gender, age, and disease severity Women valued emotional support, while men emphasised informational support	There is a need for culturally sensitive, specialised diabetes care that provides consistent support from the time of diagnosis
Wan et al. (2023) Dietary management of type 2 diabetes mellitus among South Asian immigrants: A mixed‐methods study	Investigate dietary patterns and explore barriers and facilitators to dietary management among South Asian immigrants with type 2 diabetes in Australia	Mixed‐methods study with three 24‐h dietary recalls and semi‐structured interviews Data analysed using SPSS for quantitative and NVivo for qualitative analysis	18 self‐identified South Asian immigrants with type 2 diabetes Participants were recruited from Victorian primary care clinics and social media	Australia Conducted across primary care clinics and social media platforms in Victoria, Australia	Participants had lower intake of grains, fruits, and dairy than Australian dietary recommendations Reported difficulties in adapting dietary advice into their routines and lacked knowledge of available support Relied more on health professionals than on family or friends for support	Improving access to organisational support and adapting dietary recommendations into daily routines are essential for better diabetes management among South Asian immigrants
Leung et al. (2014) Health literacy issues in the care of Chinese American immigrants with diabetes: a qualitative study	Investigate why Chinese American immigrants with diabetes struggle with health literacy despite available resources	Qualitative Focus groups and interviews with thematic analysis.	29 first‐generation Chinese immigrants with type 2 diabetes, aged ≥ 45	USA Community health centres and a retirement village in Los Angeles, California	Identified cultural, structural, and personal barriers affecting health literacy, such as respect for authority, reluctance to burden others, and lack of health insurance	Health literacy issues in Chinese immigrants require culturally sensitive approaches that address specific cultural norms and barriers
Hadziabdic et al. (2020) Development of a group‐based diabetes education model for migrants with type 2 diabetes, living in Sweden	Develop a culturally appropriate diabetes education model for migrants with type 2 diabetes in Sweden	Literature review, previous studies, and collaboration with a multi‐professional diabetes team to create a focus group‐based education model	Migrants with type 2 diabetes living in Sweden	Sweden Primary healthcare centres	A five‐session, group‐based education model focusing on individual beliefs about health and illness, facilitated by a diabetes specialist nurse with support from a multi‐professional team	The model is tailored to individual and cultural needs, aiming to improve diabetes knowledge and self‐care among migrants
McEwen et al. (2010) Type 2 Diabetes Self‐Management Social Support Intervention at the U.S.‐Mexico Border	Test the efficacy of a culturally tailored diabetes self‐management social support intervention for Mexican American adults	Quantitative Single‐group pre‐test and post‐test design A bilingual CDE and Nurse Researcher developed a culturally tailored diabetes self‐management social support intervention Six‐monthly group sessions and three home visits were conducted by community health workers, in collaboration with a Certified Diabetes Educator	21 Mexican American adults with type 2 diabetes	U.S.‐Mexico border region Arizona‐Sonora	Improved self‐management activities, diabetes knowledge, and reduced distress; no significant changes in HbA1c or BMI	Community health workers successfully delivered a culturally tailored intervention that improved self‐management behaviours, though physiological outcomes remained unchanged
Hjelm and Bard (2013) Beliefs About Health and Illness in Latin‐American Migrants with Diabetes Living in Sweden	Explore health and illness beliefs in Latin‐American migrants with diabetes in Sweden and their impact on self‐care and care‐seeking behaviour	Qualitative Qualitative exploratory study with focus group interviews	Nine Latin‐American migrants with diabetes, aged 36–77, living in Sweden	Sweden Focus groups held in secluded rooms, with participants recruited from a diabetes clinic	Health seen as freedom from disease and well‐being; reliance on both biomedical and traditional remedies; economic hardship impacts health; active self‐care with limited reliance on healthcare professionals	Culturally tailored care is essential, incorporating discussions of alternative treatments and addressing financial and migrational challenges
Joo and Lee (2016) Barriers to and facilitators of diabetes self‐management with elderly Korean‐American immigrants	Explore the barriers and facilitators of diabetes self‐management among elderly first‐generation Korean‐American immigrants with type 2 diabetes in the Midwest, USA	Qualitative Qualitative descriptive design using three focus groups (*N* = 18) and five individual interviews Data were recorded, transcribed, translated, and analysed using content‐based analysis	23 first‐generation Korean‐American elderly immigrants aged 65 years or older with type 2 diabetes for at least one year, living in the Midwest	Midwest Korean community in the Midwest	Barriers: high costs, language issues, memory loss. Facilitators: family support, time, seeking information.	Addressing barriers and enhancing facilitators can improve diabetes management for elderly Korean‐Americans
Brämberg et al. (2012) Lack of individualised perspective: A qualitative study of diabetes care for immigrants in Sweden	To describe the care provided by a diabetes nurse specialist (DNS) and the care needs expressed by immigrants with type 2 diabetes mellitus (T2DM)	Qualitative Qualitative, exploratory design using observations of clinical encounters between a DNS and 10 immigrant patients with T2DM. Data were analysed using qualitative content analysis	10 immigrants with T2DM (6 women and 4 men), originally from the Middle East, visiting the DNS at a community health centre in Sweden	Sweden A community health centre in western Sweden, serving about 7000 people, 10% of whom were foreign‐born	The study found a power imbalance in clinical encounters The DNS controlled the structure and content of the visits, limiting patient involvement. Conversations focused on medical aspects, with little attention to individual experiences or social and practical matters	The care provided by the DNS was primarily technique‐centred, lacking individualised care The study suggests shifting towards a more patient‐centred approach to improve self‐management and adherence to diabetes care
Choi et al. (2018) Optimising the effectiveness of diabetes education in an East Asian population	To explore learning behaviours of Chinese people during diabetes education and suggest tailored strategies	Qualitative Case study using observations and interviews across Australia, China, and Singapore. Data analysed using thematic analysis	39 observations and 22 interviews with health professionals and Chinese individuals with diabetes	Australia, China, and Singapore Diabetes education sessions in hospitals and community centres across three countries	Chinese participants preferred clear, prescriptive instructions, were passive learners, and sought detailed facts. Trust‐building over time facilitated behaviour change	Diabetes education should be culturally adapted with clear instructions and rapport‐building to encourage behaviour change
Staff et al. (2017) Patients requests and needs for culturally and individually adapted supportive care in type 2 diabetes patients	To compare metabolic control and supportive care requests between Nordic and non‐Nordic type 2 diabetes patients	Mixed Methods Comparative study with 184 patients attending routine check‐ups Data collected from medical records and structured interviews	151 Nordic and 33 non‐Nordic type 2 diabetes patients in a socioeconomically vulnerable area of Linköping, Sweden	Linköping, Sweden Primary healthcare centre	Non‐Nordic patients had poorer metabolic control and requested more supportive care, especially more frequent contact with nurses and help with dietary habits Fewer non‐Nordic participants (60.6%) achieved the HbA1c target compared to Nordic participants (80.1%), with a significant difference (*p* = 0.016)	Culturally and individually adapted care is not only medically necessary but also requested by non‐Nordic patients
Magny‐Normilus et al. (2019) Self‐Management of Type 2 Diabetes in Adult Haitian Immigrants: A Qualitative Study	Explore the lived experiences of adult Haitian immigrants managing type 2 diabetes in the U.S.	Qualitative Phenomenological study with individual interviews and thematic analysis	16 Haitian immigrants with type 2 diabetes, aged 40–63, living in the U.S. for an average of 11 years	USA Urban outpatient clinic in the northeastern U.S.	Four themes: self‐reliance, spirituality, nostalgia for Haiti, and desire for better patient‐provider relationships	Culturally tailored care and improved communication with healthcare providers are needed to support diabetes self‐management in Haitian immigrants
